# Tandem Versus Single Autologous Stem Cell Transplantation for High‐Risk Multiple Myeloma in the Era of Novel Agents: A Real‐World Study of China

**DOI:** 10.1002/cam4.70573

**Published:** 2025-01-02

**Authors:** Xuelin Dou, Juan Ren, Jiangtao Li, Xiaodan Liu, Li Bao, Yuan Chen, Peng Zhao, Yuping Zhong, Nan Peng, Lei Wen, Leqing Cao, Yang Liu, Daoxing Deng, Fengrong Wang, Liru Wang, Hui Liu, Xiaojun Huang, Xiaodong Mo, Jin Lu

**Affiliations:** ^1^ Peking University People's Hospital, Peking University Institute of Hematology, National Clinical Research Center for Hematologic Disease, Beijing Key Laboratory of Hematopoietic Stem Cell Transplantation Peking University Beijing China; ^2^ Department of Hematology The First Affiliated Hospital of Xi'an Jiao Tong University Shanxi China; ^3^ Department of Hematology, Beijing Hospital, National Center of Gerontology Institute of Geriatric Medicine, Chinese Academy of Medical Sciences Beijing China; ^4^ Department of Hematology The Affiliated Hospital of Qingdao University Shandong China; ^5^ Department of Hematology, Beijing Jishuitan Hospital Capital Medical University Beijing China; ^6^ Department of Hematology The Affiliated Hospital of Guizhou Medical University Guizhou China; ^7^ Department of Hematology Qingdao Municipal Hospital Qingdao China; ^8^ Department of Hematology, Fu Xing Hospital Capital Medical University Beijing China

**Keywords:** autologous stem cell transplant, multiple myeloma, tandem

## Abstract

**Background:**

This study compares the efficacy and safety of single autologous stem cell transplantation (ASCT) versus tandem ASCT for multiple myeloma (MM) patients in the era of novel agents.

**Methods:**

A total of 112 high‐risk MM patients were included (single ASCT, *(n* = 57) or tandem ASCT*(n* = 55) in this retrospective multicenter study. Responses and outcomes were evaluated.

**Results:**

At 100 days after ASCT1 and ASCT2, 36 (63.2%) versus 45 (81.8%) patients achieved sCR/CR, 16 (28.1%) versus 7 (12.7%) patients achieved VGPR, and 5 (8.8%) versus 1 (1.8%) patient achieved PR, respectively, in the single and tandem ASCT cohorts. The 3‐year cumulative incidence of non‐relapse mortality and disease progression was 0% versus 7.3% (*p* = 0.083), and 45.8% versus 25.8% (*p* = 0.039), respectively, for the single and tandem ASCT cohort. The tandem ASCT cohort showed a trend of better 3‐year probability of PFS (58.1% vs. 64.7%, *p* = 0.064) compared with the single ASCT cohort. In multivariate analysis, ultra high‐risk and achieving<VGPR response after ASCT1 were associated with an inferior PFS. Ultra high‐risk was also associated with an inferior OS.

**Conclusions:**

Tandem ASCT demonstrated improved outcomes compared to single ASCT in high‐risk MM patients receiving triplet or quadruplet induction and maintenance therapy. However, patients with ultra high‐risk cytogenetics may require innovative therapeutic approaches, as tendem ASCT does not overcome their adverse prognosis.

## 1 Introduction

Over the past decades, the global incidence of multiple myeloma (MM), which ranks as the second most common hematologic malignancy, has increased and reached nearly 2 per 100,000 people worldwide [1, 2]. Even in the era of widespread use of novel agents (e.g., proteasome inhibitors [PIs] and immunomodulatory agents [IMiDs]) as induction therapy, autologous stem cell transplantation (ASCT) continues to be upheld as the cornerstone consolidation therapy for newly diagnosed transplant‐eligible MM patients. When integrated with maintenance therapy, this contemporary treatment paradigm has demonstrably improved progression‐free survival (PFS) [3, 4, 5]. However, relapse remains inevitable for the majority of patients, with reported PFS ranging from 47.2 to 67.5 months according to several randomized clinical trials (RCTs) [3, 5, 6, 7]. Particularly, for high‐risk patients who account for 15%–20% of all MM patients, the expected overall survival (OS) was less than 3 years [8].

Before the introduction of novel agents, several studies investigated the efficacy of tandem ASCT in high‐risk MM patients [9, 10, 11, 12, 13, 14]. For example, Moreau et al. [14] observed the improvements in both event‐free survival and OS in the tandem ASCT cohort compared with the single ASCT cohort in high‐risk MM patients. However, the meta‐analysis of Kumar et al. [12] and Naumann‐Winter et al. [13] showed that the survival of tandem ASCT were not superior to that of the single ASCT.

Nowadays, with the widely use of novel agents and maintenance therapy, ASCT remains irreplaceable, but the role of tandem ASCT in high‐risk MM was still controversial. In the BMT CTN 0702 trial [15], wherein 55% of patients received RVD induction therapy and 24% were classified as high‐risk, the tandem ASCT cohort showed no superiority in PFS and OS compared with the single ASCT cohort. However, long‐term follow‐up of this study suggested that high‐risk MM patients may still benefit from tandem ASCT [16]. Cavo et al. reported tandem ASCT could improve PFS and OS, especially in high‐risk patients [17]. In the EMN02/HOVON95 study [18], only the patients with del(17p) could benefit from tandem ASCT, which was similar to the results of the PETHEMA/GEM study [19]. In the study of Duggan P et al. [20], including over 400 patients with high‐risk cytogenetics, only the patients without maintenance therapy could benefit from tandem ASCT. Thus far, the role of tandem ASCT in high‐risk MM patients remains unclear in the era of using novel agents in induction and maintenance therapy after ASCT.

In this multicenter study, we aimed to identify the efficacy and safety of tandem ASCT versus single ASCT for high‐risk MM patients in the era of novel agents and maintenance therapy in a real‐world setting.

## 2 Materials and Methods

### 2.1 Patients

**FIGURE 1:**
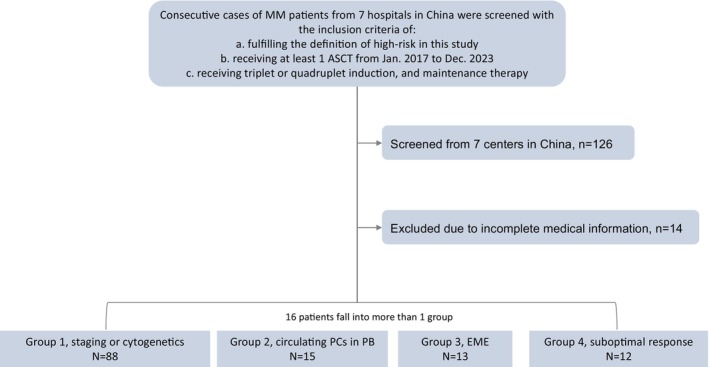
Inclusion of patients from multicenter in China.

This is a multicenter, retrospective cohort study based on the transplant database of Peking University Institute of Hematology (PUIH), First Affiliated Hospital of Xi'an Jiaotong University, Beijing Hospial, The Affiliated Hospital of Qingdao University, Beijing Jishuitan Hospital, The Affiliated Hospital of Guizhou Medical University and Qingdao Municipal Hospital. Consecutive cases of MM patients fulfilling the definition of high‐risk in this study and receiving at least one ASCT as upfront therapy from January 2017 to December 2023 were screened. Patients receiving triplet or quadruplet induction, and maintenance therapy after ASCT were included, while those with incomplete medical information were excluded (Figure 1). The study was approved by the institutional review board of Peking University People's Hospital (2024PHB173‐001) and was conducted in accordance with the *Declaration of Helsinki*.

### 2.2 Definition of High‐Risk of MM Patients

The definitions of high‐risk MM were as follows:

1. Group 1, staging or cytogenetics: presence of two or more of the following factors at diagnosis: International Staging System (ISS) stage 3, elevated dehydrogenase (LDH), gain or amplification of chromosome 1q21 (1q21+), del(17p), t(4;14), t(14;16), or hypodiploidy karyotype. Particularly, a subgroup of ultra high‐risk patients is defined as the presence of two or more of the high‐risk cytogenetic abnormalities including 1q21+, del(17p), t(4;14) or t(14;16).
2. Group 2, circulating plasma cells (PCs) in peripheral blood (PB): presence of PCs in the PB smears at diagnosis with a percentage ≤ 4% (a percentage ≥ 5% was classified as plasma cell leukemia and thus excluded);
3. Group 3, extramedullary extraosseous disease (EME): presence of extraosseous extramedullary disease at diagnosis;
4. Group 4, suboptimal response: defined as achieving only a stable disease (SD) as the maximum therapeutic response to the triplet combination by the third cycle of induction therapy.

### 2.3 Treatment Regimen

All enrolled patients underwent triplet or quadruplet induction therapy, comprising bortezomib and dexamethasone combined with one or two other classes of novel agents, or chemotherapy. The induction regimen included VRD (bortezomib‐lenalidomide‐dexamethasone), VCD (bortezomib‐cyclophosphamide‐dexamethasone), VTD (bortezomib‐thalidomide‐dexamethasone), PDD (bortezomib‐liposomal doxorubicin‐dexamethasone), and daratumumab‐based quadruplet regimen. Daratumumab‐based quadruplet regimens included included DVCD (daratumumab‐bortezomib‐cyclophosphamide‐dexamethasone), as well as alternating treatment of V‐DECP (bortezomib‐dexamethasone‐etoposide‐cyclophosphamide‐cisplatin) and Dara‐VPD (daratumumab‐bortezomib‐pomalidomide‐ dexamethasone) according to an investigator‐initiated trial [21].

After the induction phase, all the patients underwent stem‐cell mobilization. ASCT was later applied after 4–6 cycles of induction therapy. The conditioning regimen for ASCT was with melphalan at a full dose of 200 mg/m^2^, or a reduced dose of 100–140 mg/m^2^ in patients with a creatinine clearance of lower than 40 mL/min. The choice of single or tandem ASCT was according to local center practice. For patients receiving tandem ASCT, a second transplant was performed within 6 months of the first transplant. Maintenance were applied in all of the patients till disease progression, while the regimen were tailored according to patients' risk stratification, drug availability, and insurance policies specific to each center. Maintenance treatment included thalidomide, lenalidomide, bortezomib, ixazomib, and daratumumab.

### 2.4 Diagnosis, Responses and Monitoring Protocols

Patients diagnosed with MM meet the International Myeloma Working Group (IMWG) diagnostic criteria 2014 [22]. Responses were assessed according to the IMWG response criteria [23]. Extramedullary disease was classified into bone‐related masses (EMB) or extraosseous disease (EME). The response post‐ASCT was evaluated at 100 days (±30 days) after ASCT.

Bone marrow aspiration was routinely performed at baseline, within 30 days prior to ASCT, and 100 days (±30 days) after ASCT. Interphase fluorescence in situ hybridization (FISH) of bone marrow were performed at baseline to detect chromosomal abnormalities (CA) using CD138‐purified PCs by magnetic activated cell sorting (MACS), as previously described [24]. All patients were analyzed for 1q21+, del (17p), and IgH rearrangement using gene locus‐specific probes(GLP) including GLP 1q21, GLP P53, GLP IgH. If an IgH rearrangement was identified, dual‐color and dual‐fusion translocation probes such as IgH‐FGFR3, IgH‐MAF and IgH‐CCND1 were used for the detection of t(4;14)(p16;q32), t(14;16)(q32;q23) and t(11;14)(q13;q32). Three (gain) or ≥ 4 (amp) copies of 1q21 were grouped together and indicated as 1q21 + .

Minimal residual disease (MRD) was assessed by multiparameter flow cytometry (MFC) panel of CD38/CD138/CD45/CD19/CD56/CD117/cytoplasmic kappa (cκ)/cytoplasmic lambda (cλ). If patients received daratumumab treatment within 3 months prior to FCM test, additional panel of CD38/CD229/CD45/CD19/CD56/CD117/ cκ / cλ was tested. The number of cells detected was 1 × 10^6^ at a sensitivity threshold of 10^−4^.

### 2.5 Data Collection

Investigators at each hospital utilized the institutional electronic medical records of clinical databases to obtain the required information. The collected data included the information on patient demographics, diagnosis, treatment and response before ASCT, transplant regimen, MRD status before and after first ASCT (ASCT1) and second ASCT (ASCT2), maintenance therapy, and clinical outcomes. The data were independently reviewed by two physicians with sufficient experience in MM and ASCT to ensure the accuracy of the results.

### 2.6 Definition

The primary end points was PFS (survival period without progression disease after first ASCT), and the secondary endpoints inculded overall response (ORR, included CR, VGPR, and PR), OS (time from fisrt ASCT to death from any cause), and non‐relapse mortality (NRM, time from first ASCT to death from any cause without recurrence of MM). Patients who were alive without relapse were censored at last contact. The last follow‐up time was April 2024.

### 2.7 Statistical Analysis

Descriptive statistics were used to summarize covariates. The *χ*2 or Fisher's exact tests were used for categorical variables, whereas a nonparametric test was used for continuous variables. Data were censored at the time of death or last available follow‐up. Survival functions were estimated using the Kaplan–Meier method and were compared by the log‐rank test. Cox proportional hazards regression models were used to evaluate factors associated with survival and relapse. Clinical variables of age, sex, EMD, BM infiltration of plasma cell, cytogenetics, staging, and whether the application of tandem were included in univariate COX analysis. Tandem or single ASCT and co‐variates with *p* < 0.2 in univariate analyses were included in multivariate analyses and selected using a backward elimination process to fit a Cox regression model. All reported P values are two‐sided and considered significant at an overall significance level of 5%. All statistical analyses and graphing were performed with SPSS 26.0 software (SPSS, Chicago, IL) and R version 4.3.1 (R Core Team, Vienna, Austria).

## 3 Results

### 3.1 Patient Characteristics and Treatment

A total of 112 MM patients were enrolled in the analysis, with 57 and 55 receiving single and tandem ASCT, respectively (Table 1). The median age of patients in tandem ASCT cohort was significantly younger than that of the single ASCT. Ninety‐eight patients (87.5%) received triplet regimen. Fourteen patients (12.5%) received daratumumab‐based quadruplet regimen. For high‐risk profile, most patients were categorized into Group 1 (*n* = 88, 78.6%). In Group 4, 11 out of the 12 patients had their regimen intensified by incorporating additional novel agents. Sixteen (14.3%) out of the 112 patients received a reduced dose of melphalan during condition in ASCT1, and 35 (63.6%) out of the 55 patients during ASCT2. The median follow up was 30.6 (95% CI: 23.8, 37.4) months for all patients since ASCT1.

**TABLE 1:** Characteristics of patients by single and tandem ASCT cohorts.

Variables	Overall	Single ASCT	Tandem ASCT	*P*
	(*N* = 112)	(*N* = 57)	(*N* = 55)	
Median age, median years (range)	55 (25, 69)	56 (25, 69)	53 (31, 65)	0.020
Male, *n* (%)	63 (56.3%)	36 (63.2%)	27 (49.1%)	0.190
Hemoglobin < 100 g/L, *n* (%)	55 (49.1%)	30 (52.6%)	25 (45.5%)	0.568
Platelet < 100 × 10^9/L, *n* (%)	9 (8.0%)	7 (12.3%)	2 (3.6%)	0.162
eGFR < 60 mL/min/1.73 m^2^, *n* (%)	30 (26.8%)	17 (29.8%)	13 (23.6%)	0.599
LDH>upper normal limit, *n* (%)	13 (11.6%)	6 (10.5%)	7 (12.7%)	0.945
M protein type, *n* (%)				
IgG	56 (50.0%)	27 (47.4%)	29 (52.7%)	0.584
IgA	26 (23.2%)	16 (28.1%)	10 (18.2%)	
IgD	8 (7.1%)	3 (5.3%)	5 (9.1%)	
Bi‐clonal	2 (1.8%)	1 (1.8%)	1 (1.8%)	
Light chain	18 (16.1%)	8 (14.0%)	10 (18.2%)	
Non‐secretory	2 (1.8%)	2 (3.5%)	0 (0%)	
Extramedullary disease, *n* (%)				0.065
EMB	23 (20.5%)	7 (12.3%)	16 (29.1%)	
EMS	13 (11.6%)	6 (10.5%)	7 (12.7%)	
BM infiltration of plasma cell ≥ 60%, n (%)	23 (20.5%)	15 (26.3%)	8 (14.5%)	0.191
Hypodiploidy karyotype, *n* (%)	8 (7.1%)	4 (7.0%)	4 (7.3%)	1.000
1q21+, *n* (%)	70 (62.5%)	35 (61.4%)	35 (63.6%)	0.961
del (17p), *n* (%)	29 (25.9%)	13 (22.8%)	16 (29.1%)	0.587
t (4;14), *n* (%)	44 (39.3%)	24 (42.1%)	20 (36.4%)	0.668
t (14;16), *n* (%)	8 (7.1%)	6 (10.5%)	2 (3.6%)	0.272
Ultra‐high risk, *n* (%)	50 (44.6%)	29 (50.9%)	21 (38.2%)	0.177
ISS disease stage, *n* (%)				
I	20 (17.9%)	9 (15.8%)	11 (20.0%)	0.489
II	30 (26.8%)	18 (31.6%)	12 (21.8%)	
III	62 (55.4%)	30 (52.6%)	32 (58.2%)	
R‐ISS disease stage, n (%)				
I	14 (12.5%)	8 (14.0%)	6 (10.9%)	0.634
II	44 (39.3%)	24 (42.1%)	20 (36.4%)	
III	54 (48.2%)	25 (43.9%)	29 (52.7%)	
R2‐ISS disease stage, *n* (%)				
Low risk	8 (7.1%)	4 (7.0%)	4 (7.3%)	0.949
Low‐intermediate	12 (10.7%)	7 (12.3%)	5 (9.1%)	
Intermediate‐high	54 (48.2%)	26 (45.6%)	28 (50.9%)	
High	38 (33.9%)	20 (35.1%)	18 (32.7%)	
Induction regimen, *n* (%)				
RVD	53 (47.3%)	23 (40.4%)	30 (54.5%)	0.151
VCD	25 (22.3%)	16 (28.1%)	9 (16.4%)	
PDD	11 (9.8%)	6 (10.5%)	5 (9.1%)	
VTD	9 (8.0%)	7 (12.3%)	2 (3.6%)	
Daratumumab‐based quadruplet	14 (12.5%)	5 (8.8%)	9 (16.4%)	
Group 1, staging or cytogenetics, *n* (%)	88 (78.6%)	43 (75.4%)	45 (81.8%)	0.554
Group 2, circulating PCs in PB, *n* (%)	15 (13.4%)	11 (19.3%)	4 (7.3%)	0.094
Group 3, EME, *n* (%)	13 (11.6%)	6 (10.5%)	7 (12.7%)	0.945
Group 4, suboptimal response, *n* (%)	12 (10.7%)	8 (14.0%)	4 (7.3%)	0.361

Abbreviations: eGFR, estimated glomerular filtration rate; EMB, extramedullary bone‐related disease; EME, extramedullary extraosseous disease; ISS, International Staging System; LDH, lactate dehydrogenase; PB, peripheral blood; PCs, plasma cells; PDD, bortezomib‐liposomal doxorubicin‐dexamethasone; R‐ISS, revised ISS; R2‐ISS, second revised ISS; RVD, bortezomib‐lenalidomide‐dexamethasone; VCD, bortezomib‐cyclophosphamide‐dexamethasone; VTD, bortezomib‐thalidomide‐dexamethasone.

### 3.2 Responses

The overall response rate (ORR) was 93.8% prior to ASCT including 19 (17.0%) patients in sCR, 18 (16.1%) patients in CR, 35 (31.3%) in VGPR and 33 (29.5%) in PR in the total cohort. At 100 days after ASCT1, 79 (70.5%) patients achieved sCR/CR in the total cohort, with 36 (63.2%) and 43 (78.2%) in the single and tandem ASCT cohort, respectively (*p* = 0.469). In addition, 24 (21.4%) patients achieved VGPR in the total cohort, with 16 (28.1%) and 8 (14.5%) in the single and tandem ASCT cohort, respectively (*p* = 0.160). Eight (7.1%) patients achieved PR in the total cohort, with 5 (8.8%) and 3 (5.5%) in the single and tandem ASCT cohort, respectively (*p* = 0.526). One (1.8%) patients achieved SD after ASCT1 in the tandem cohort.

**FIGURE 2:**
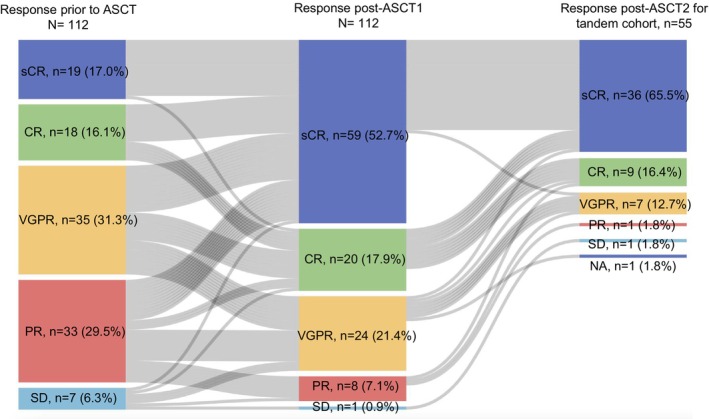
The Sankey diagram shows the flow of responses prior to ASCT and post‐ASCT1 for the total cohort, as well as post‐ASCT2 for the tandem cohort.

In the tandem ASCT cohort, 45 (81.8%) patients achieved sCR/CR (sCR, *n* = 36; CR, *n* = 9) after ASCT2. In addition, 7 (12.7%), and 1 (1.8%) patients achieved VGPR and PR respectively after ASCT2. One patient remained SD, and 1 died of NRM 2 weeks after ASCT2 without response evaluation (Figure 2).

**FIGURE 3:**
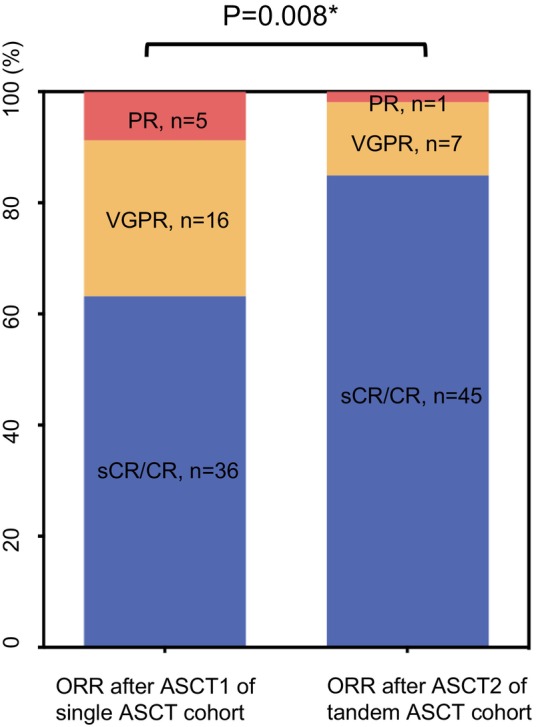
The overall response rates (ORR) between post‐ASCT1 of single ASCT cohort and post‐ASCT2 of tandem ASCT cohort (data normalized to 100%). *The Chi‐Square test for trend indicated a significant difference in the ORR rate between the two groups.

The ratio of sCR/CR after ASCT1 and ASCT2 was 63.2% and 81.9%, for single and tandem ASCT cohort, respectively. The ratio of VGPR after ASCT1 and ASCT2 was 28.1% and 12.7%, for single and tandem ASCT cohort, respectively. The ratio of PR after ASCT1 and ASCT2 was 8.8% and 1.8%, for single and tandem ASCT cohort, respectively. The overall response rate (i.e., CR/sCR + VGPR+PR) after ASCT2 of tandem ASCT cohort was better than that after ASCT1 of single ASCT cohort (*p* = 0.008, Figure 3).

### 3.3 NRM and Disease Progression

**FIGURE 4:**
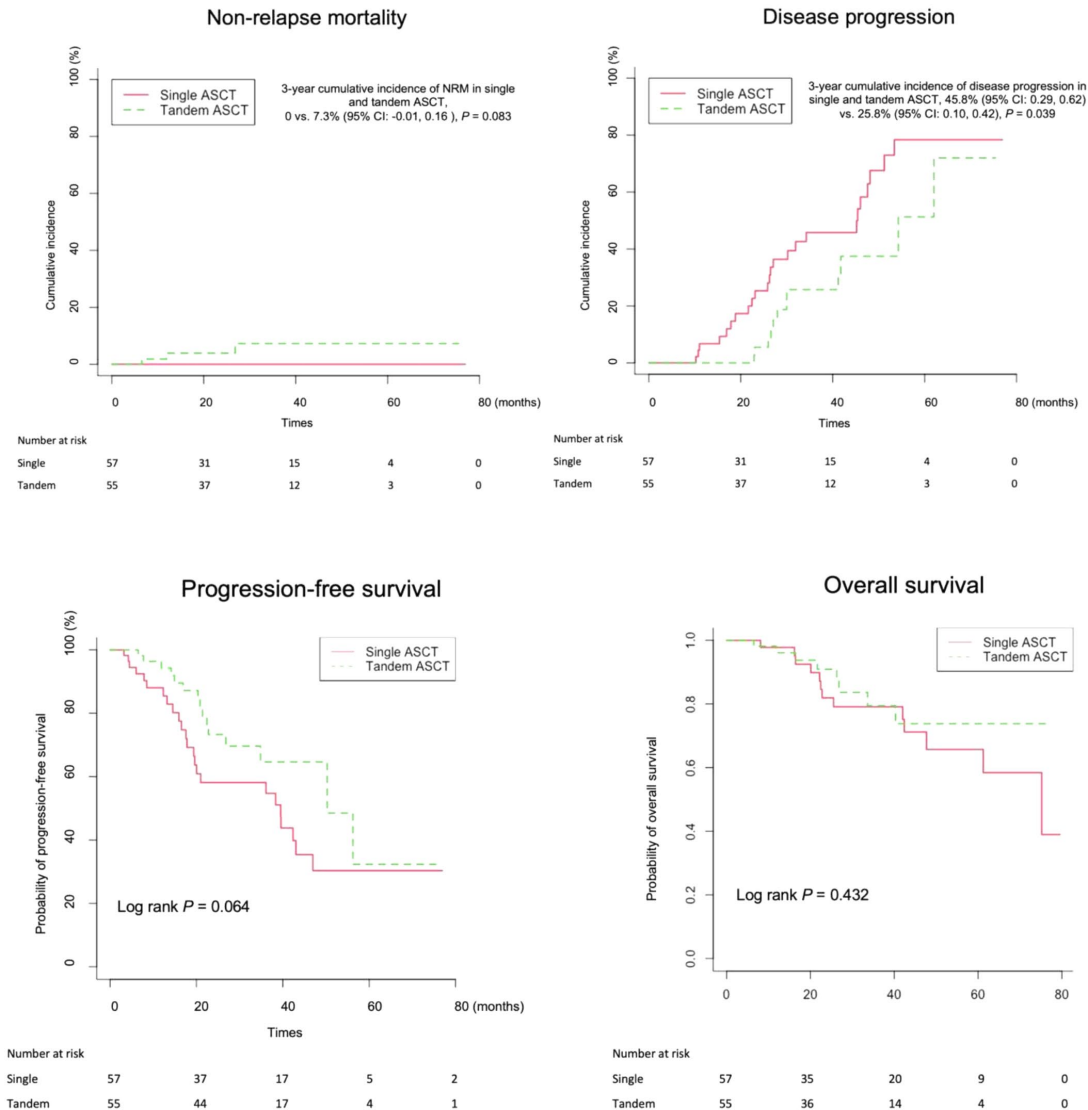
Cumulative incidence of non‐relapse mortality and disease progression, kaplan–meier curve of progression‐free survival and overall survival for single and tandem ASCT cohorts.

No NRM occurred in the single ASCT cohort, while three patients (5.5%) in the tandem ASCT cohort died from NRM after ASCT2. The 3‐year cumulative incidence of NRM was 0 vs. 7.3% (95% confidence interval [CI]: 0, 0.16), for single and tandem ASCT cohort, respectively (*p* = 0.083, Figure 4).

Disease progression occurred in 36 (32.1%) patients, with 24 (42.1%) and12 (21.8%) patients in the single and tandem ASCT cohort, respectively. The 3‐year cumulative incidence of disease progression was 45.8% (95% CI: 0.29, 0.62) versus 25.8% (95% CI: 0.10, 0.42) for single and tandem ASCT cohort, respectively (*p* = 0.039, Figure 4).

### 3.4 Progression‐Free Survival

At the last follow‐up, disease progression or death occurred in 24 (42.1%) and 15 (27.3%) patients in single and tandem ASCT cohort, respectively. The median PFS for the whole cohort was 43.0 (95% CI: 34.3, 51.7) months, with 39.5 (95% CI: 17.8, 61.2) months and 50.3 (95% CI: 30.3, 70.2) months in the single and tandem ASCT cohort (*p* = 0.064), respectively (Figure 4). For the total cohort, the 3‐year probability of PFS was 62.7% (95% CI: 0.53, 0.75), with 58.1% (95% CI: 0.45, 0.76) and 64.7% (95% CI: 0.50, 0.84) in the single and tandem cohort, respectively.

The median PFS for Group 1, 2, 3, and 4 were 39.6 (95% CI: 34.4, 44.7), 22.8 (95% CI: 20.6, 25.0), 56.2 (95% CI: 10.7, 101.8), and not reached, respectively. There was a trend of a superiority of better PFS in tandem ASCT compared with single ASCT in both group 1 and group 4 (Figure S1).

The detailed results of univariate analysis were showed in Table S1. HGB < 100 g/L, PLT < 100 × 10^9/L, ultra high‐risk, tandem ASCT and response post‐ASCT1 were included in multivariable analysis. Ultra high‐risk (HR 2.3, 95% CI 1.19–4.55, *p* = 0.013) and achieving<VGPR response at post‐ASCT1 (HR 4.37, 95% CI 1.57–12.15, *p* = 0.005) were associated with a pooer PFS in multivariable analysis. Tandem ASCT was not assoicated with a better PFS in multivariate analysis.

### 3.5 Overall Survival

At the last follow‐up, 21 (18.8%) patients died, with13 (22.8%) and 8 (14.5%) and patients died in the single and tandem ASCT cohort, respectively. The median OS for the total cohort was not reached, with 75.2 (95% CI, 50.7, 99.7) and not reached in the single and tandem cohort (*p* = 0.432, Figure 4). For the total cohort, the 3‐year probability of OS was 79.7% (95% CI: 0.71, 0.90), with 79.1% (95% CI: 0.67, 0.93) and 79.5% (95% CI: 0.66, 0.95), respectively, in the single and tandem cohort.

The median OS for group 1 was 75.2 (95% CI: 39.9, 110.5), and not reached for all other groups including group 2, 3 and 4.

HGB < 100 g/L, PLT < 100 × 10^9/L, EMD, ultra high‐risk, tandem ASCT and response post‐ASCT1 were included in multivariate COX analysis (Table S2). Ultra high‐risk was the only independent factor associated with inferior OS (HR 5.8, 95% CI 1.92–17.45, *p* = 0.002). Tandem ASCT was not assoicated with a superior OS in multivariate analysis.

## 4 Discussion

In this multicenter study, we compared clinical outcomes between single and tandem ASCT in 112 high‐risk MM patients who received triplet or quadruplet induction and maintenance after ASCT. The 3‐year cumulative incidence of NRM and disease progression was 0 versus 7.3%, and 45.8% versus 25.8%, respectively, for single and tandem ASCT cohort. In addition, we observed that tandem ASCT cohort showed a trend of better 3‐year probability of PFS compared with single ASCT cohort nevertheless the 3‐year probability of OS was comparable between the cohorts. In multivariate analysis, ultra high‐risk and achieving<VGPR response at post‐ASCT1 were associated with a poorer survival. Thus far, this is one of the largest studies which compared the clinical outcomes between single and tandem ASCT in MM patients with a comprehensive high‐risk criteria.

Many studies had compared the efficacy of single versus tandem ASCT in high‐risk MM patients [9, 10, 11, 12, 13, 14, 25]. In the era of novel agents, several studies also identified the efficacy of tandem ASCT in high‐risk MM patients [17, 18, 19], but the results were inconsistent. One explanation was that the criteria of “high‐risk” were different among these studies. For example, Villalba et al. [19] defined high‐risk MM as having at least 1 one of the following cytogenetic abnormalities: del (17p), t(4;14), t(14;16) or gain of 1q21 (≥ 4 copies). Stadtmaue et al. [16] defined high‐risk MM by the presence of elevated β2‐microglobulin, or presence of cytogenetic abnormalities, including t(4;14), t(14;20), t(14;16), del (17p). While in studies of Duggan et al. [20] and Cavo et al. [18], high‐risk was defined as the presence of del (17p), t (4;14), or t (14;16). Additionally, Gagelmann et al. [26] included t(14;20), gain(1q), or del(1p) in their definition of high‐risk cytogenetics. According to Zamagni et al. [8], high‐risk patients should be characterized by by factors which could significanlty reduce survival including tumor biology and suboptimal responses to therapy. In order to include as thorough as possible of patients with high‐risk characteristics for relapse, we included 4 groups of high‐risk MM patients in the present study. Thus far, our study used the more comprehensive definition of high‐risk criteria, which might help to further compare the efficacy between single and tandem ASCT in these patients.

In addition, Suzuki et al. [27] reported that the 30‐month rate of PFS was only 65% and 57.4% for those with PR and SD before ASCT. Similarly, we observed that patients who showed poor response to induction therapy (i.e., group 4 of patients achieving only SD by the third cycle of triplet induction) was only 30% in single ASCT cohort. What's more, we observed that the 3‐year probability of PFS of tandem ASCT cohort was superior to single ASCT cohort in group 4, which suggested that these patients might benefit from tandem ASCT.

We also explored the effcacy of tandem ASCT in the patients with the “ultra high‐risk” category, characterized by the presence of two (“double‐hit”) or three (“triple‐hit”) cytogenetic abnormalities including 1q21+, del(17p), t(4;14) or t(14;16). We observed that tandem ASCT could not further improve the survival of these patients, which was similar to the results of PETHEMA/GEM study [19]. In multivariate analysis, ultra high‐risk was independently associated with poored PFS and OS, indicating that tandem ASCT could not overcome the adverse prognosis of ultra high‐risk. This subgroup of patients might need more innovative management.

It was well‐accpepted that circulating PCs in PB and presence of EMS were both adverse factors for PFS [28, 29, 30, 31, 32]; however, no study had compared the efficacy between single and tendem ASCT in these patients. We observed that the 3‐year probabilities of PFS and OS were comparable between single and tandem ASCT in both group 2 and group 3. It might be due to the relatively small samples of these subgroups in the present study and we could not further idnetify the efficacy of tandem ASCT in these patients. It was worthy of comparing the efficay of single and tandem ASCT in these subgroups in future.

Our study was limited by its retrospective nature, and relatively small population, especially for group 4 of patients with suboptimal responses. In addition, the follow‐up period was relatively short in the present study; however, we had observed that tandem ASCT cohort showed significant lower probability of disease progression and a trend of better PFS compared with single ASCT, and we speculated the benefit might be more prominent with a longer follow‐up. The heterogeneity of induction and maintenance regimens is another limitation of this study. However, this exactly reflects the complexity in real world setting, that is, in this rapidly shifting of MM treatment pattern with more and more risk‐adapted strategies, it is difficult to homogenize all regimens. Last but not the least, in the era of novel agents, induction therapy should contain at least two of the three classes of novel agents: PIs, IMiDs or CD38 antibody. However, only 60% of patients in our study received at least two classes. A prospective study with a larger cohort and homogeneous treatment regimens is needed to confirm the findings of our real‐world analysis.

## 5 Conclusions

For high‐risk MM patients, upfront tandem ASCT seemed to be better than single ASCT in patients receiving triplet or quadruplet induction and maintenance therapy. However, patients with ultra high‐risk may not benefit from tandem ASCT and more innovative therapy strategies should be identified in these patients.

## Author Contributions

**Xuelin Dou:** data curation, investigation, resources, writing – original draft, conceptualization, methodology; **Juan Ren:** data curation, investigation, validation, formal analysis, resources, writing – original draft; **Jiangtao Li:** data curation, investigation, validation, formal analysis, resources, writing – original draft; **Xiaodan Liu:** data curation, investigation, validation, resources. **Li Bao:** data curation, resources. **Yuan Chen:** data curation, resources. **Peng Zhao:** data curation, resources. **Yuping Zhong:** data curation, formal analysis, resources. **Nan Peng:** data curation, formal analysis, resources. **Lei Wen:** data curation, resources. **Leqing Cao:** data curation, resources, methodology. **Yang Liu:** data curation, resources, validation, conceptualization, methodology. **Daoxing Deng:** data curation, resources. **Fengrong Wang:** data curation, resources, supervision, project administration, writing – review and editing. **Liru Wang:** data curation, resources, writing – review and editing. **Hui Liu:** data curation, resources. **Xiaojun Huang:** supervision, project administration. **Xiaodong Mo:** visualization, supervision, project administration, funding acquisition, writing – review and editing. **Jin Lu:** formal analysis, supervision, project administration, funding acquisition, visualization, writing – review and editing, conceptualization.

## Ethics Statement

The study complies with all ethics regulations. This study was reviewed and approved by the institutional review board of Peking University People's Hospital, with the approval number 2024PHB173‐001.

## Consent

All participants provided written informed consent to participate in the study.

## Conflicts of Interest

The authors declare no conflicts of interest.

## Supporting information


Data S1.

## Data Availability

Available from the corresponding author on reasonable request.
