# Supplementary material for: Tandem Versus Single Autologous Stem Cell Transplantation for High‐Risk Multiple Myeloma in the Era of Novel Agents: A Real‐World Study of China
Source: Cancer Med. 2025 Jan 2;14(1):e70573. doi: 10.1002/cam4.70573 (PMC11694139; doi:10.1002/cam4.70573)
Supplement: Supplementary file 1 — Data S1. [file CAM4-14-e70573-s001.docx]

**Supplemental Figure S1**

| 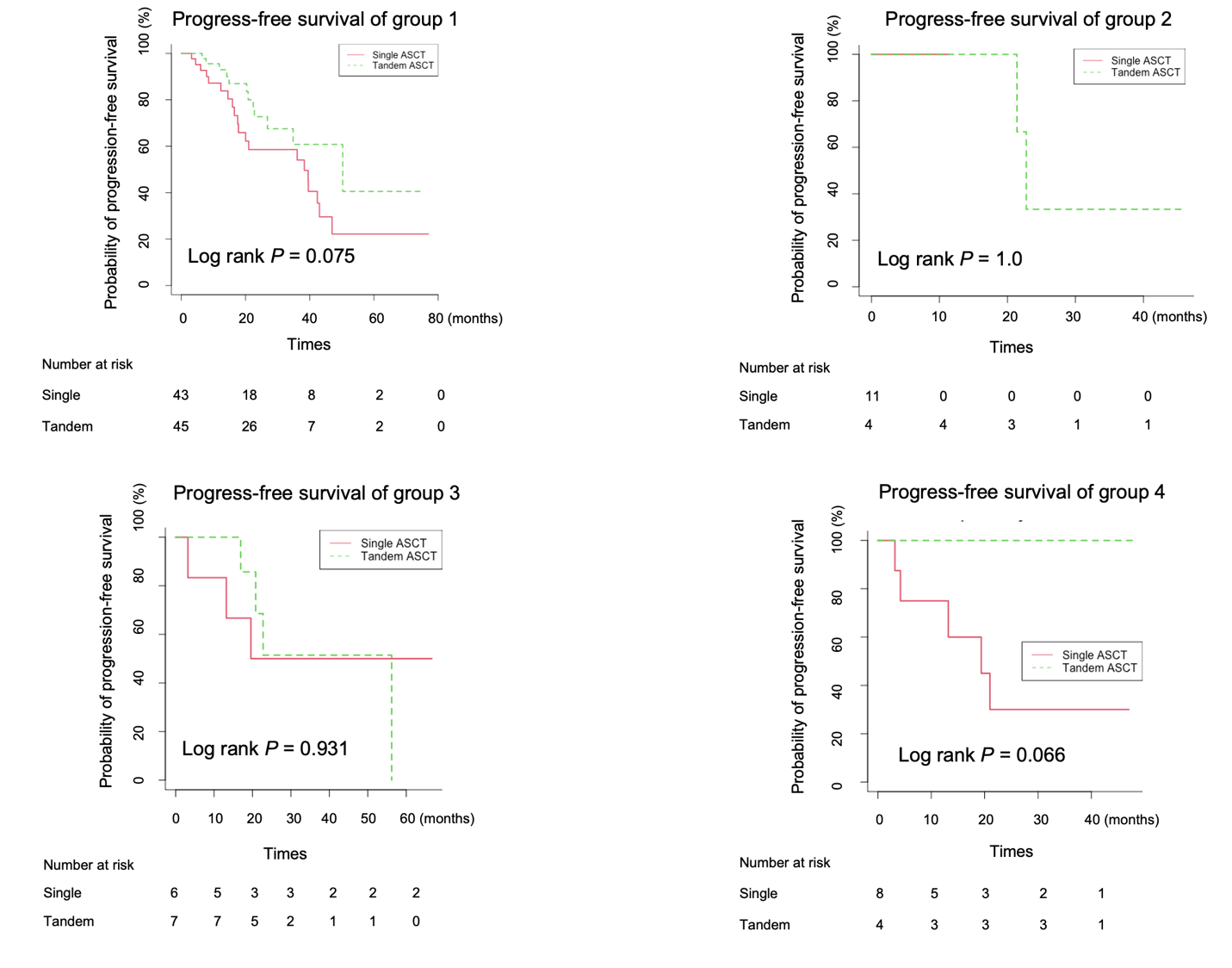 |
| --- |
| Figure S1. Kaplan-Meier curve of progression-free survival for the single and tandem ASCT cohort in each group. |

**Supplemental Table S1.** Univariate and multivariate Cox analysis of progression-free survival.

| Variables | Univariate analysis | | Multivariate analysis | |
| --- | --- | --- | --- | --- |
|  | HR (95% CI) | P value | HR (95% CI) | P value |
| Age (continuous) | 1.01 (0.97 to 1.05) | 0.656 |  |  |
| Male sex | 0.80 (0.43 to 1.52) | 0.501 |  |  |
| Extramedullary disease |  | 0.645 |  |  |
| EMB vs. none | 1.43 (0.64 to 3.20) | 0.385 |  |  |
| EME vs. none | 1.26 (0.54 to 2.96) | 0.594 |  |  |
| BM infiltration of plasma cell ≥ 60% | 0.94 (0.42 to 2.14) | 0.889 |  |  |
| 1q21+ | 1.84 (0.92 to 3.67) | 0.082 |  |  |
| del(17p) | 1.59 (0.79 to 3.20) | 0.197 |  |  |
| t(4;14) | 1.01 (0.54 to 1.90) | 0.979 |  |  |
| t(14;16) | 2.02 (0.48 to 8.52) | 0.340 |  |  |
| Ultra-high risk | 2.07 (1.09 to 3.94) | 0.027 | 2.33 (1.19 to 4.55) | 0.013 |
| R2-ISS disease stage |  | 0.371 |  |  |
| Low-intermediate vs. low | 0.59 (0.12 to 2.92) | 0.513 |  |  |
| Intermediate-high vs. low | 0.84 (0.24 to 2.86) | 0.775 |  |  |
| High vs. low | 1.42 (0.41 to 4.92) | 0.580 |  |  |
| Induction regimen |  | 0.882 |  |  |
| VCD vs. VRD | 0.83 (0.39 to 1.76) | 0.619 |  |  |
| PDD vs. VRD | 0.85 (0.25 to 2.90) | 0.799 |  |  |
| VTD vs. VRD | 0.98 (0.33 to 2.90) | 0.971 |  |  |
| Daratumumab-based quadruplet vs. VRD | 0.47 (0.11 to 2.02) | 0.310 |  |  |
| Group 1, staging or cytogenetics vs. other | 1.49 (0.65 to 3.38) | 0.346 |  |  |
| Group 2, circulating PCs in PB vs. other | 0.96 (0.23 to 4.01) | 0.951 |  |  |
| Group 3, EME vs. other | 1.17 (0.51 to 2.69) | 0.707 |  |  |
| Group 4, suboptimal response vs. other | 1.37 (0.53 to 3.53) | 0.511 |  |  |
| Tandem ASCT vs. single ASCT | 0.55 (0.29 to 1.05) | 0.068 |  |  |
| Response at post-ASCT1 |  | 0.072 |  | 0.030 |
| CR vs. sCR | 0.99 (0.41 to 2.37) | 0.977 | 0.85 (0.35 to 2.07) | 0.721 |
| VGPR vs. sCR | 1.40 (0.62 to 3.15) | 0.413 | 1.23 (0.54 to 2.79) | 0.617 |
| PR/SD vs. sCR | 3.69 (1.35 to 10.08) | 0.011 | 4.37 (1.57 to 12.15) | 0.005 |

**Supplemental Table S2.** Univariate and multivariate Cox analysis of overall survival.

| Variables | Univariate analysis | | Multivariate analysis | |
| --- | --- | --- | --- | --- |
|  | HR (95% CI) | P value | HR (95% CI) | P value |
| Age (continuous) | 0.99 (0.94 to 1.04) | 0.677 |  |  |
| Male sex | 0.79 (0.33 to 1.86) | 0.587 |  |  |
| Extramedullary disease |  | 0.146 |  |  |
| EMB vs. none | 2.02 (0.75 to 5.39) | 0.163 |  |  |
| EME vs. none | 0.29 (0.04 to 2.21) | 0.232 |  |  |
| BM infiltration of plasma cell ≥ 60% | 1.82 (0.7 to 4.69) | 0.218 |  |  |
| 1q21+ | 3.49 (1.17 to 10.42) | 0.025 |  |  |
| del(17p) | 2.1 (0.84 to 5.23) | 0.112 |  |  |
| t(4;14) | 1.48 (0.62 to 3.51) | 0.379 |  |  |
| t(14;16) | 2.17 (0.29 to 16.45) | 0.452 |  |  |
| Ultra-high risk | 6.68 (2.23 to 20.01) | 0.001 | 5.79 (1.92 to 17.45) | 0.002 |
| R2-ISS disease stage |  | 0.193 |  |  |
| Low-intermediate vs. low | 11400.43(0 to Inf) | 0.936 |  |  |
| Intermediate-high vs. low | 22341.83(0 to Inf) | 0.931 |  |  |
| High vs. low | 52362.05 (0 to Inf) | 0.926 |  |  |
| Induction regimen |  | 0.878 |  |  |
| VCD vs. VRD | 1.09 (0.41 to 2.89) | 0.866 |  |  |
| PDD vs. VRD | 0.64 (0.81 to 5.10) | 0.677 |  |  |
| VTD vs. VRD | 0.39 (0.05 to 3.10) | 0.372 |  |  |
| Daratumumab-based quadruplet vs. VRD | 1.19 (0.25 to 5.56) | 0.826 |  |  |
| Group 1, staging or cytogenetics vs. other | 6.16 (0.83 to 45.93) | 0.076 |  |  |
| Group 2, circulating PCs in PB | 0.05 (0 to 183.72) | 0.464 |  |  |
| Group 3, EME | 0.25 (0.03 to 1.88) | 0.178 |  |  |
| Group 4, suboptimal response | 1.07 (0.25 to 4.67) | 0.928 |  |  |
| Tandem ASCT vs. single ASCT | 0.70 (0.29 to 1.71) | 0.434 |  |  |
| Response at post-ASCT1 |  | 0.213 |  |  |
| CR vs. sCR | 1.68 (0.49 to 5.76) | 0.406 |  |  |
| VGPR vs. sCR | 2.91 (1.05 to 8.09) | 0.040 |  |  |
| PR/SD vs. sCR | 2.61 (0.54 to 12.75) | 0.235 |  |  |
